# Practice of Traditional Medicine and Associated Factors Among Residents in Eastern Ethiopia: A Community-Based Cross-Sectional Study

**DOI:** 10.3389/fpubh.2022.915722

**Published:** 2022-06-14

**Authors:** Shambel Nigussie, Abduro Godana, Abdi Birhanu, Tilahun Abdeta, Fekade Demeke, Magarsa Lami, Kabtamu Gemechu, Addis Eyeberu, Kasahun Bogale, Deribe Bekele Dechasa, Tamirat Getachew, Abraham Negash, Siraj Aliyi, Fentahun Meseret, Haregeweyn Kibret, Bekelu Berhanu Nigatu, Gebisa Dirirsa, Tilahun Bete Gebremariam, Kefelegn Bayu, Yideg Abinew, Dawud Wedaje Ali, Fenta Wondimneh, Ayichew Alemu, Jemal Husen Dilebo, Addisu Alemu, Yadeta Dessie

**Affiliations:** ^1^School of Pharmacy, College of Health and Medical Science, Haramaya University, Harar, Ethiopia; ^2^School of Medicine, College of Health and Medical Science, Haramaya University, Harar, Ethiopia; ^3^School of Nursing and Midwifery, College of Health and Medical Sciences, Haramay University, Harar, Ethiopia; ^4^College of Medicine and Health Science, Jigjiga University, Jigjiga, Ethiopia; ^5^School of Medical Laboratory Science, College of Health and Medical Sciences, Haramaya University, Harar, Ethiopia; ^6^Department of Environmental Health, College of Health and Medical Sciences, Haramaya University, Harar, Ethiopia; ^7^School of Public Health, College of Health and Medical Sciences, Haramaya University, Harar, Ethiopia

**Keywords:** traditional medicine, practice, utilization, Harari, Eastern Ethiopia

## Abstract

**Introduction:**

Although the Practice of traditional medicine is accorded great importance worldwide, it seems to face a notable challenge. A notable challenge is the lack of a reference standard for determining the appropriate utilization of traditional medicine for patients. There is little evidence about the usual utilized traditional medicine in the study area. Therefore, this study aimed to assess practice of traditional medicine and associated factors among residents in Eastern Ethiopia.

**Methods:**

A community-based cross-sectional study was conducted from January 20, 2022 to February 20, 2022. About 818 study participants were recruited using a systematic random sampling technique. Data were collected by face-to-face interviews. The collected data were analyzed using Statistical Package for Social Sciences (SPSS) version 22 computer software. The association between an outcome variable and independent variables was assessed using binary logistic regression and the strength of association was presented using Adjusted Odd Ratio (AOR) with its 95% confidence intervals (CI).

**Result:**

A total of 803 participants were included in the final analysis with a 98.2 % response rate. From the total study participants, 563 (70.1%) [95%CI: 66.8–73.3] had used traditional medicine in the past 6 months. Factors such as being farmer [AOR = 1.06; 95%CI: (1.03–3.7)], having a diploma degree or higher [AOR = 3.2, 95% CI (1.4–7.3)] and having no history of chronic disease [AOR = 0.21; 95% CI: (0.1–0.5)] were significantly associated traditional medicine practice.

**Conclusion:**

The proportion of traditional medicine practice was high. The most commonly utilized traditional medicines were Damakase, Tenadam, Zingibil and Erate. A national health policy should give a great emphasize on rational utilization of traditional medicine.

## Introduction

Traditional Medicine (TM) encompasses a variety of health practices, approaches, knowledge, and beliefs that include herbal, animal, and/or mineral-based medicines, spiritual therapies, manual techniques, and exercises, used singly or in combination to treat, diagnose, or prevent disease (1). The rise in the use of traditional medicine led to a multinational enterprise of traditional health practice. Billions of dollars are spent annually on traditional medicine in various developed countries. For example, around $32 billion was spent on traditional medicine in the United States in 2012, which is expected to increase to $60 billion by 2021 (2). The World Health Organization (WHO) estimates that the global traditional medicine market is worth US$83 billion per year (3).

Countries in Africa, Asia, and Latin America use traditional medicine to meet some of their primary health care needs (4–6). Traditional eye medicine is a traditional medicine common in Africa. Between 13.2 and 82.3% of the population used it in Africa (7–9).

Despite high emphasis given to the utilization of traditional medicine around the world, it appears to face major challenges (10). A notable challenge is the lack of a reference standard for determining the appropriate practice of traditional medicine for patients. Another notable challenge is the lack of a national policy to manage and legalize the use of traditional medicine. These significant problems have led to the creation of incorrect and incomplete information on traditional medicine (11).

In Ethiopia, there is hardly an urban or rural area where traditional medicine is not involved in health care, as it is an integral part of the local culture and easily accessible to the majority of the population (12). Although traditional medicine is widespread, it is not practiced consistently or similarly in Ethiopia. Utilizers of traditional medicine are quite diverse and differ significantly between regions (13). The standard guidelines and regulations for traditional medicine are not as strict (14, 15).

In Ethiopia, a few cross-sectional community-based studies have identified some critical factors in the practice of traditional medicine. Some of the reported factors were age, education, occupation, cultural beliefs, monthly income, and religious beliefs (16–18). However, the evidence is not strong enough to show which of these determinants are directly linked to traditional medicine practice. This is because the studies to date have been inconsistent and the associated factors influencing the practice of traditional medicine vary within the region. Additionally, the previous study was conducted only in the urban area of Eastern Ethiopia (19). Therefore, this study aimed to assess practice of traditional medicine and associated factors in Eastern Ethiopia.

## Methods and Materials

### Study Area, Design and Period

A community-based cross-sectional study was conducted in the Harari region of eastern Ethiopia. The region is located 526 km from east of Addis Ababa, the capital city of Ethiopia. It is completely surrounded by the regional state of Oromia. According to the 2007 national central statistical agency report, the population size of Harari region is 183,415, and the region is subdivided into 36 localities (Kebeles), which hold ~46,815 households and 101,539 population with the age of 18 years and above (20). The study was conducted from January 20, 2022 to February 20, 2022.

### Source and Study Population

All households in the Harari Regional State were the source population of the study. All head of households in selected kebeles were the study population.

### Inclusion and Exclusion Criteria

Being head of households and living in the region for at least 6 months were the inclusion criteria of this study. Head of households who had mental illness during data collection were excluded.

### Sample Size Determination

The sample size was calculated by using the single population proportion formula (*n* = (Zα/2)^2^p (1–p)/d^2^), where n is the minimum sample size required, p is the estimated proportion of traditional medicine practice, and Zα/2 is the value of the standard score at 95% confidence interval (1.96); the assumptions of confidence level are at 95% = 1.96, a margin of error (d) = 0.05, and design effect = 2. For this study, *p* = 59.2% (the proportion of traditional medicine practice) was used (18). The number of households included in the study was calculated as


$$n=2^{*}z2\times p\left(1-p\right)/d2$$

Thus, n= 2(1.96)2 x 0.592(1–0.592)/ (0.05)2 =743

Including 10% contingency (non-response rate) which is 75, the sample size was 818. Therefore, the final sample size for this study was 818.

### Sampling Techniques

**Figure 1:**
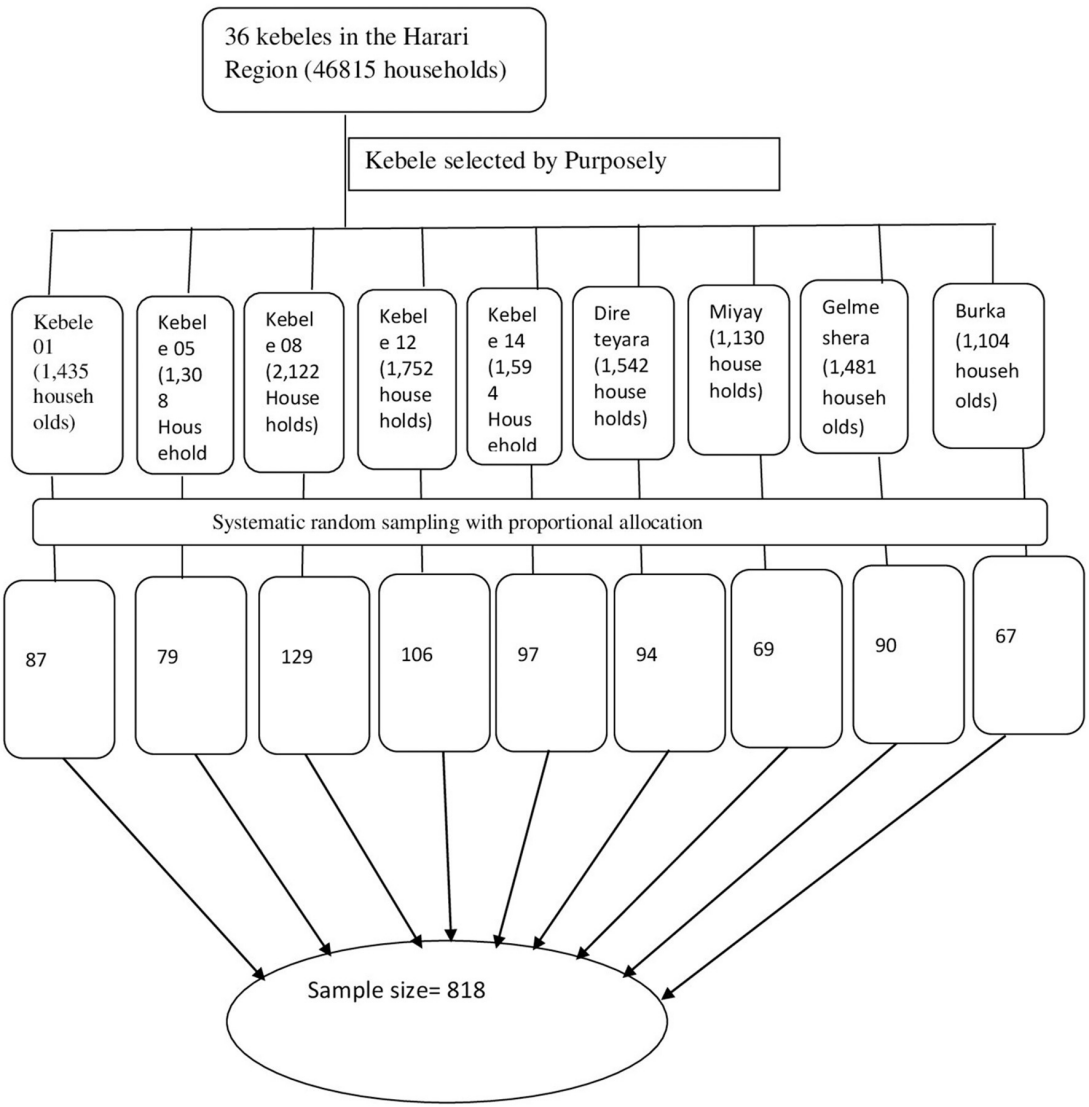
Multistage sampling which shows the sampling procedure to draw the study participants from source population.

A multi-stage sampling method was used to select the study participants. There are 19 urban and 17 rural kebeles in the study area. From these 36 kebeles, nine kebeles were selected by purposely. Then, from the selected Kebeles, 818 households were allocated proportionately. Each household was selected using a systematic random sampling procedure, and then the head of household within the selected household was enrolled in the study (Figure 1).

### Data Collection Method

A semi-structured questionnaire was used to collect all the required data through a face-to-face interview. The questionnaire was prepared in English by reviewing different literatures and translated into the local language (Amharic and Afan Oromo) as well-back translated to English and includes the following variables: age, gender, place of residence, level of education, religion, occupation, marital status, existence of a chronic illness, access to pharmacy, family health care profession, income, utilization, type of traditional medicine, side effect, intervention, and outcome. Data were collected by 10 BSc nurses under the guidance of six MSC-holder supervisors.

### Data Quality Control

To ensure the quality of the data, the questionnaire was pre-tested in haramaya woreda prior to data collection. All errors found during the pre-test were corrected and changes were made in the final version of the data collection questionnaire. The data collectors were trained prior to the data collection process. The well-trained supervisor monitored and checked to ensure the completeness and consistency of the data. All collected data was checked for completeness and consistency during data management, storage and analysis.

### Operational Definitions

Traditional Medicine Practice: In this study, the participant was said to have traditional medicine practice when he/she had used one of the following traditional medicines at least once in the past 6 months to treat a disease: Tenadam, Nech Shinkurt, Kebericho, Moringa, Lomi, Dama Kese, Zingibil, Feto, Kebericho, Nech bahirzaf, Senafitch, Besobila, Tosegn, Tikur azmud, Qunda berbere, Abish, Qarafa, Dimbelal, Irate, Girawa, Buna, Feto and others.

### Data Analysis and Presentation

The collected data were entered into the computer using Epi Data Statistical Software version 3.1 and then exported to Statistical Packages for Social Sciences (SPSS) version 22 for coding, editing, cleaning and analysis. Continuous variables were described using mean and standard deviation (SD), while frequency and percentage were used for categorical variables. A bivariable logistic regression analysis was performed to select candidate variables for the multivariable logistic regression model. Accordingly, variables with a *P* ≤ 0.25 in bivariable logistic regression were entered into the multivariable logistic regression analysis to control for potential confounding variables. A multivariable logistic regression analysis was used to determine the association between the independent and outcome variables. The presence and strength of statistically significant associations was indicated at *p* < 0.05 and using AOR with its 95% confidence intervals (CI). Finally, the data were presented using text and tables.

### Ethical Consideration

An ethical clearance letter was obtained from the Haramaya University, College of Health and Medical Sciences, Institutional Health Research Ethics Review Committee (reference number: IHRERC/017/2022). Official letter of cooperation to conduct the study was sent to the Administrator of the Harari Regional State Health Bureau. Prior to data collection, informed, voluntary, written and signed consent was obtained from participants. The participants were also informed that the information received from the participants will be kept strictly confidential. Throughout the study, COVID-19 safety measures (wearing a mask, maintaining social distancing, and using alcohol-based hand sanitizer) were applied to protect participants, data collectors as well as supervisors from the deadly pandemic.

## Results

### Sociodemographic Characteristics of the Participants

A total of 818 participants were recruited to participate in this study. Of these, 803(98.2%) participants answered the interview in full and included it in the final analysis. The mean age of the participants was 41.05 years with a standard deviation (SD) of 15.36. Majority of the participants 494 (61.5%) were women. Sixty percent of the participants lived in rural areas. More than half of study participants 429 (53.4%) were married. Almost 45% of the participants (43.5%) were farmers. Ten percent of participants 76 (9.5%) had a history of chronic disease. More than two-thirds of participants 546 (68%) had access to the pharmacy. There were healthcare professionals in the family of 162 (20.2%) of the participants. The average monthly income of 374 (46.6%) participants was <1,000 ETB (Table 1).

**Table 1:** Socio-demographics characteristics of the participants in Harari region, Easter Ethiopia, 2022 (*n* = 803).

**Characteristics**	**Frequency**	**Percentage**
Sex of the respondent		
Female Male	494 309	61.5 38.5
Age (years) category		
18–27 28–37 38–47 48–57 58–67	209 167 196 125 74 32	26.0 20.8 24.4 15.6 9.2 4.0
hspace*5ptunderline>68		
Residence of the respondents		
Urban Rural	314 489	39.1 60.9
Educational level		
Unable to read and write Able to read and write Primary school Secondary school Diploma and above	188 150 191 188 86	23.4 18.7 23.8 23.4 10.7
Marital status of the respondents		
Single Married Widowed Divorced Separated	128 429 171 46 29	15.9 53.4 21.3 5.7 3.6
Occupation status of the respondents		
Housewife Farmer Merchant Government employee	221 349 179 54	27.5 43.5 22.3 6.7
Religion of the respondents		
Muslim Orthodox Protestant Catholic	391 275 112 25	48.7 34.2 13.9 3.1
Presence of chronic illness		
Yes No	76 727	9.5 90.5
Access to Pharmacy
Yes No	257 546	32 68
Presence of health professional in their family		
Yes No	162 641	20.2 79.8
Average monthly income (ETB)		
<1,000 1,000–2,000 >2,000	374 149 280	46.6 18.6 34.9

### Traditional Medicine Practice

The prevalence of traditional medicine practice was 70.1% [95%CI: 66.8–73.3]. Of 563 participants who used traditional medicine, 335(59.5%) participants used it for themselves. Around 308 (54.7%) participants who took traditional medicine encountered side effects of this medicine. More than half of the study participants 332 (59%) reported as they were permanently cured from their disease when they used traditional medicine (Table 2).

**Table 2:** Traditional medicine utilization in Harari region, Eastern Ethiopia, 2022.

**Characteristics**	**Frequency**	**Percentage**
Traditional medicine utilization		
Yes No	563 240	70.1 29.9
For whom you used traditional medicine most commonly		
Your self Your wife Your child	335 126 102	59.5 22.3 18.1
Who advised you to take TM?		
Family Friend Healthcare practitioner Traditional medicine healer Elderly	70 78 100 99 113	15.2 16.9 21.7 21.5 24.5
Have you encountered any adverse effects from it?		
Yes No	308 255	54.7 45.3
What was that side/adverse effect you encountered?		
Diarrhea Headache Abdominal pain Nausea Vomiting Skin rash	38 58 52 58 45 57	12.3 18.8 16.8 18.8 14.6 18.5
Measures taken for your encountered side effect		
Going to health facilities Stopping the medicine	217 91	70.4 29.5
Have you taken TM with MD?		
Yes No	460 343	57.3 42.7
The outcome of traditional medicine you used for the management of illness?		
Permanent cure Symptomatic relief	332 231	59.0 41.0

*TM, traditional medicine; MM, modern medicine*.

### Traditional Medicine Utilized by Study Participants

*Damakase* was the most commonly used traditional medicine, used by 295 (36.7%) participants to treat the common cold and tonsillitis. *Zingibil* (25.9%), *Erate* (22.7%) and *Feto* (17.2%) were used by study participants to treat abdominal cramps (Table 3).

**Table 3:** Traditional medicine used by study participants in Harari region, Eastern Ethiopia, 2022.

**Local name**	**Scientific name**	**Disease treated**	**Frequency**	**Percentage**
Damakase	Ocimum lamiifolium	Common cold and tonsillitis	295	36.7
Tenadam	Ruta chalepensis	Common cold	276	34.4
Zingibil	Zingiber officinale	Abdominal cramp	208	25.9
Erate	Aloe megalacantha	Abdominal cramp	182	22.7
Senafich	Brassica nigra	Common cold	140	17.4
Nechshinkurt	Allium sativum	Common cold and gastritis	139	17.3
Feto	Lepidium sativum	Abdominal cramp	138	17.2
Nechibahrzaf	Eucalpytus globulus	Common cold	137	17.1
Lomi	Citrus aurantifolia	Scabies	129	16.1
Tosegn	Thymus schimperi	Hypertension and common cold	52	6.4
Kebericho	Echinops kebericho	Malaria	33	4.1
Buna	Coffea arabica	Infected wound and cough	32	3.9

### Factors Associated With Traditional Medicine Practice

In the bivariate analysis, variables with a *P* < 0.25 (educational level, occupation, access to pharmacy, income, history of chronic diseases) were moved into a multivariable model. In the multivariable logistic regression analysis, educational level, occupation, and history of chronic diseases were significantly associated with traditional medicine practice. Participants with a diploma degree or higher were more than three times more likely to use traditional medicines than those who were unable to read and write [AOR = 3.2, 95% CI (1.4–7.3), *p* = 0.007]. In this study, Farmers were 1.06 times more likely to use traditional medicines than participants who were government employees [AOR = 1.06, 95% CI: (1.03, 3.7), *p* = 0.001]. Participants with no history of chronic illness were 79% less likely to use traditional medicine than participants with a history of chronic illness: [AOR = 0.21; 95% CI (0.1, 0.5), *p* = 0.00] (Table 4).

**Table 4:** Multivariable analysis of factors associated with traditional medicine practice.

**Variables**	**Practice of TM**		**COR (95%CI)**	**AOR (95%CI)**	***P*-value**
	**Yes (%)**	**No (%)**			
Educational level					
Unable to read and write	131 (69.7)	57 (30.3)	1	1	(0.5–1.9)
Able to read and write	101 (67.3)	49 (32.7)	1.11 (0.7–1.8)	0.77 (0.4–1.5)	0.43
Primary school	132 (69.1)	59 (24.6)	1.02 (0.6–1.6)	1.32 (0.8–2.3)	3.2 (1.4–7.3)
Secondary school	151 (80.3)	37 (19.7)	0.56 (0.4–0.9)		0.33
Diploma and above	48 (55.8)	38 (44.2)	1.81 (1.1–3-1)	0.007*	0.96
Occupation					
Gov't employee	40 (74.1)	14 (25.9)	1	1	0.001*
Farmer	256 (73.4)	93 (26.6)	1.04 (1.01–2.2)	1.06 (1.03–3.7)	0.07
Merchant	124 (69.3)	55 (30.7)	1.27 (0.5–2.4)	1.93 (0.7–2.9)	0.29
housewife	143 (64.7)	78 (35.3)	1.56 (0.8–3.2)	2.3 (0.9–3.6)	
Access to Pharmacy					
Yes	189 (73.5)	68 (26.5)	1	1	0.90
No	374 (68.5)	172 (31.5)	1.28 (0.9–1.8)	0.99 (0.6–1.6)	
Family income (ETB)					
>2,000	205 (73.2)	75 (26.8)	1	1	0.19
1,000–2,000	107 (71.8)	42 (28.2)	1.07 (0.5–1.2)	1.08 (0.7–2.1)	0.15
<1,000	251 (67.1)	123 (32.9)	1.24 (0.7–2.3)	1.28 (0.9–2.8)	
History of chronic illness					
Yes	52 (68.4)	24 (31.6)	1	1	0.00*
No	511 (70.3)	216 (29.7)	0.92 (0.6–1.5)	0.21 (0.1–0.5)	
Self-reported outcome of TM used					
Symptomatic relief	153 (66.2)	78 (33.8)	1	1	0.53
Permanent cure	220 (66.3)	112 (33.7)	0.99 (0.7–1.4)	0.88 (0.6–1.3)	

TM, traditional medicines, *statistically significant.

## Discussion

This study was conducted to determine traditional medicine practice and its associated factors in Eastern Ethiopia. Being farmer, having a diploma degree or higher and having no history of chronic disease were significantly associated with traditional medicine practice.

The results of this finding showed that overall practice of traditional medicine in the community accounted for 70.1%, which was lower than the study conducted in Shopa Bultum, Jimma Town and Nekemte Town (21–23) and similar to the study, which was shown in the communities of Merawi Town and Uganda (18, 24). The discrepancy may be due to differences in cultural acceptance, perceived efficacy against certain types of disease, physical accessibility, and affordability of traditional medicine vs. modern medicine (24, 25).

In the current study, a higher proportion of traditional medicine practice were reported among study participants compared to previous study conducted in the city of Harar (19). This could be due to the earlier study conducted only in Harar city and with a small sample size.

Around 35.3% of participants had information about the use of traditional medicine for different disease from traditional medicine healer which was differ from source of information for Communities of Debre Tabor Town (26) and the most common disease for which the participants used those traditional medicines were common cold and tonsilitis. The users of traditional medicine are somewhat diverse and significantly differ between regions (13).

In present study, the most commonly utilized traditional medicine was damakase (36.7%) which is not in lined previous study report (19). This difference could be due to differences in the age and educational level of the study participants. Knowledge of traditional medicine is good among the elderly and is related to educational level (26).

In this study, 59% of participants reported as they were permanently cured from their disease when they used traditional medicine, while another study in the city of Jimma showed that 83.4% of the participants were symptomatically relieved of their disease. The lack of a reference standard for determining the appropriate dosage of traditional medicine for patients can lead to discrepancies in the treatment outcome of the illness (11).

In this study, higher level of education was associated with higher use of traditional medicine. This contradicts reports from previous studies where utilizers of traditional medicine had little or no formal education (27–31), but similar results were reported in the towns of Enugu and Debre Tabor (26, 32). People with a high level of education may have more knowledge and opportunities to take care of themselves than people with a lower level of education. In the current study, the use of traditional medicine may reflect a greater focus and concern about health-related issues among those with higher education than a preference for the type of health care.

Participants who had the occupation of farmer showed a significant difference (AOR = 1.06, CI = 1.03–3.7) regarding the practice of traditional medicine compared to other participant groups. This finding was comparable to the study conducted in north-western Ethiopia, which indicates an association of occupation with the utilization of traditional medicine (18). Participants with a history of chronic illness showed a significant difference (AOR = 0.21, CI = 0.1–0.5) in terms of practice of traditional medicine compared to participants with a history of chronic illness.

### Limitation of Study

In this study factors associated with traditional medicine practice were assessed using across-sectional design, which might not show causal relationships with potential factors.

## Conclusion

The prevalence of traditional medicine practice was high. The most commonly utilized traditional medicines were Damakase, Tenadam, Zingibil and Erate. Being farmer, having a diploma degree or higher and having no history of chronic disease were the most important factors influencing on practice of traditional medicine.

### Recommendations

The majority of the participants have used traditional medicine in the past 6 months. However, a number of participants used a single traditional medicine for different types of diseases. So, a national health policy should give a great emphasize on rational utilization of traditional medicine. More than half of the study participants 332 (59%) reported as they were permanently cured from their disease when they used traditional medicine. This is a potential area of research to develop effective drugs to treat diseases that cannot be treated with currently available modern medicines, so further research on this idea should be encouraged.

## Data Availability Statement

The original contributions presented in the study are included in the article/supplementary material, further inquiries can be directed to the corresponding author.

## Ethics Statement

The studies involving human participants were reviewed and approved by Haramaya University, College of Health and Medical Sciences, Institutional Health Research Ethics Review Committee (reference number: IHRERC/017/2022). The Ethics Committee waived the requirement of written informed consent for participation.

## Author Contributions

SN and AG conceived the idea and contributed to data analysis. AB, TA, FD, ML, KG, AE, KBo, DD, TGet, AN, SA, FM, HK, BN, GD, TGeb, KBa, YA, DA, FW, AyA, JD, AdA, and YD contributed to data review, data analysis, drafting, and revising final draft. All authors read and approved the final version of the article to be published and agreed on all aspects of this work.

## Conflict of Interest

The authors declare that the research was conducted in the absence of any commercial or financial relationships that could be construed as a potential conflict of interest.

## Publisher's Note

All claims expressed in this article are solely those of the authors and do not necessarily represent those of their affiliated organizations, or those of the publisher, the editors and the reviewers. Any product that may be evaluated in this article, or claim that may be made by its manufacturer, is not guaranteed or endorsed by the publisher.
